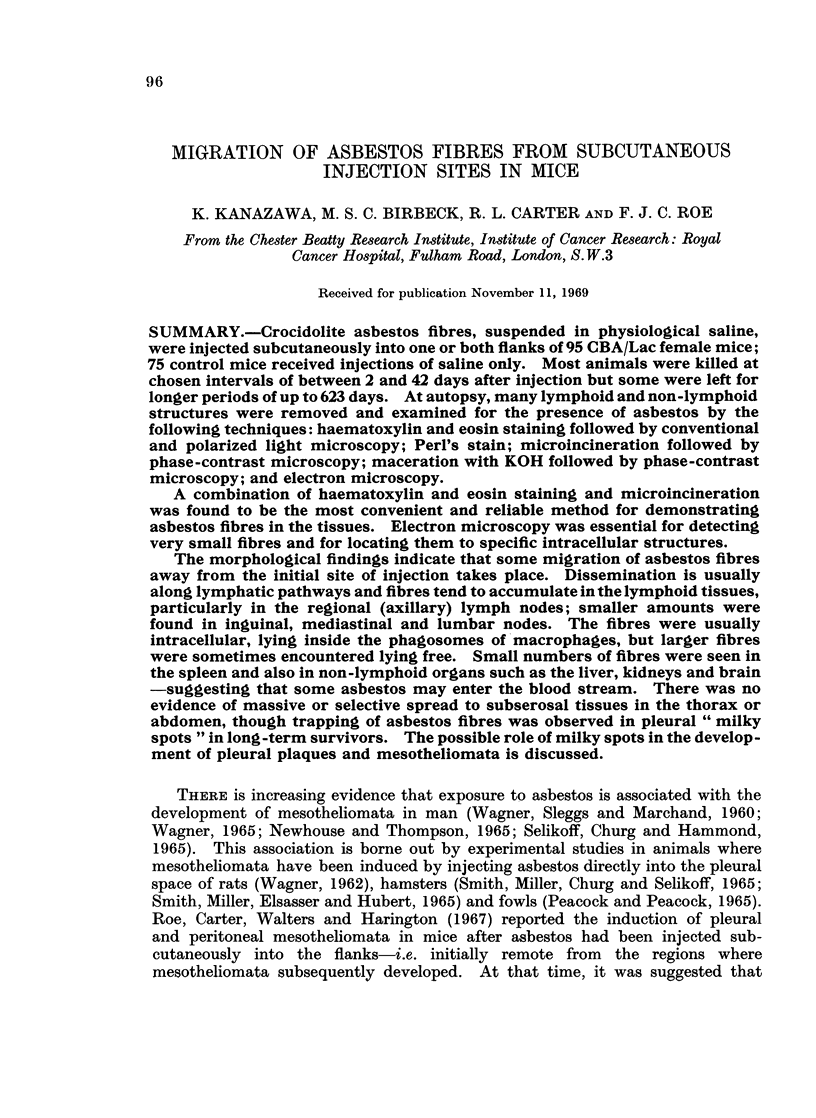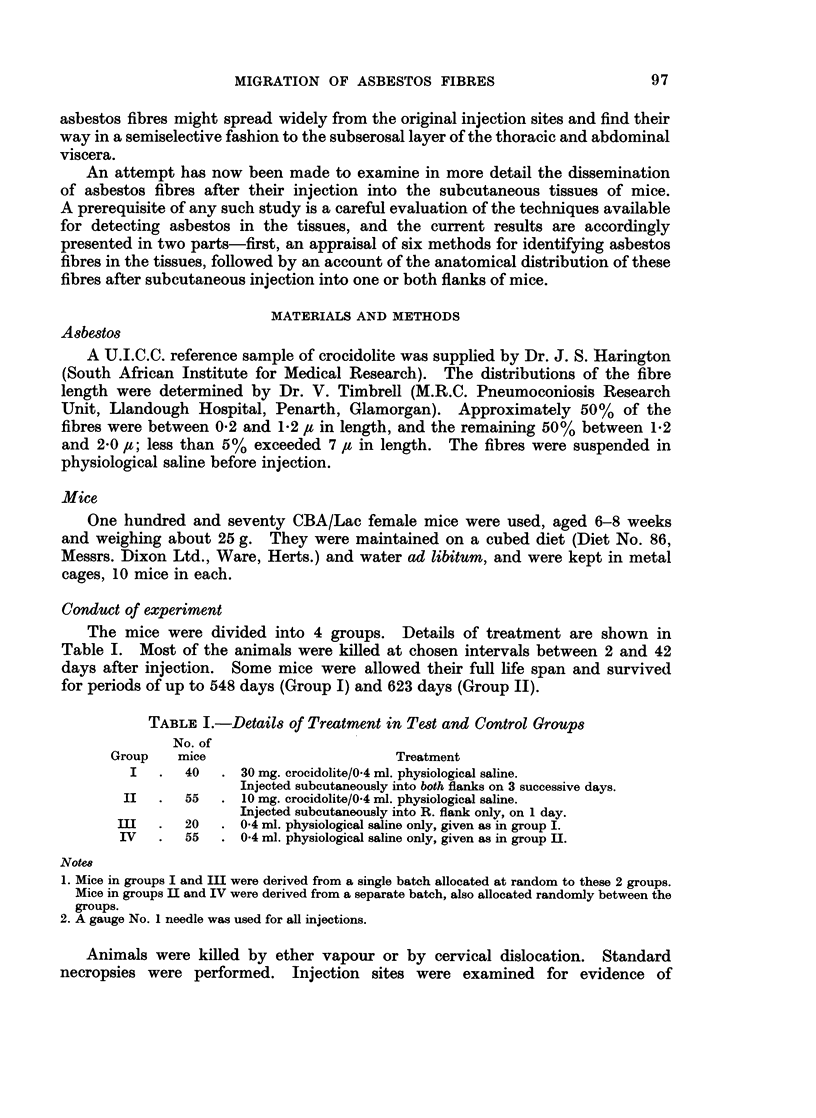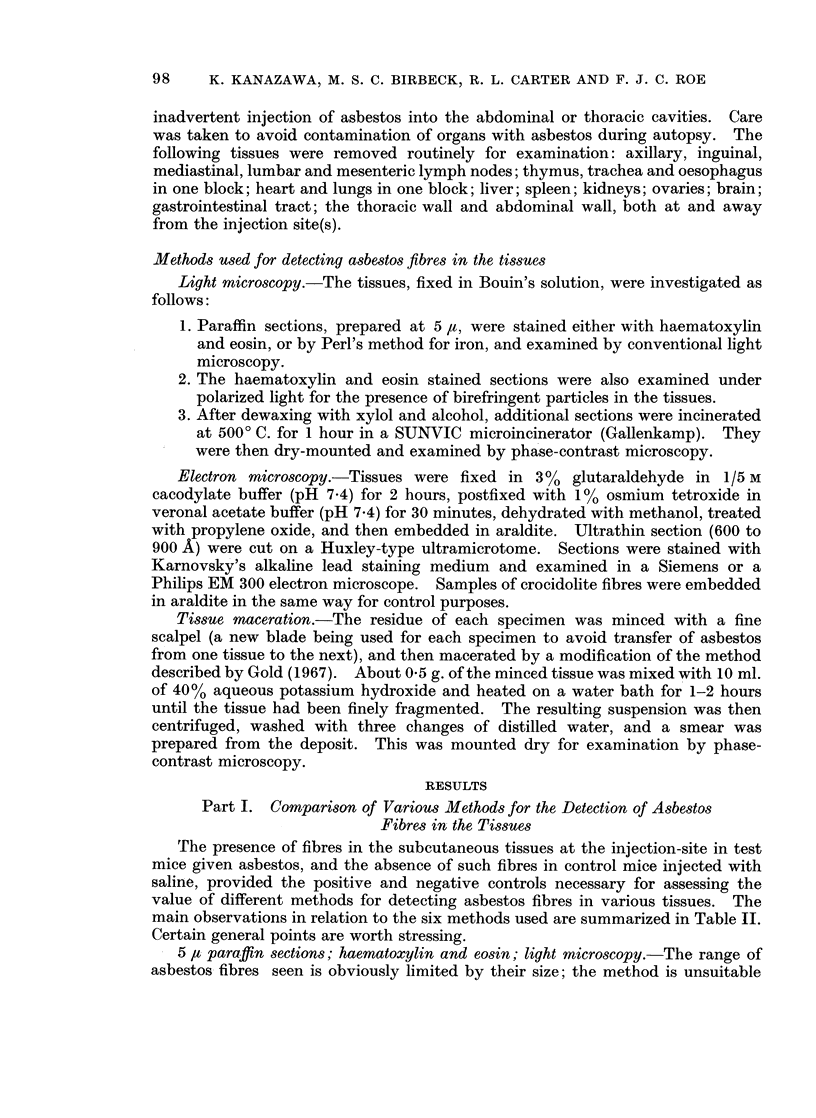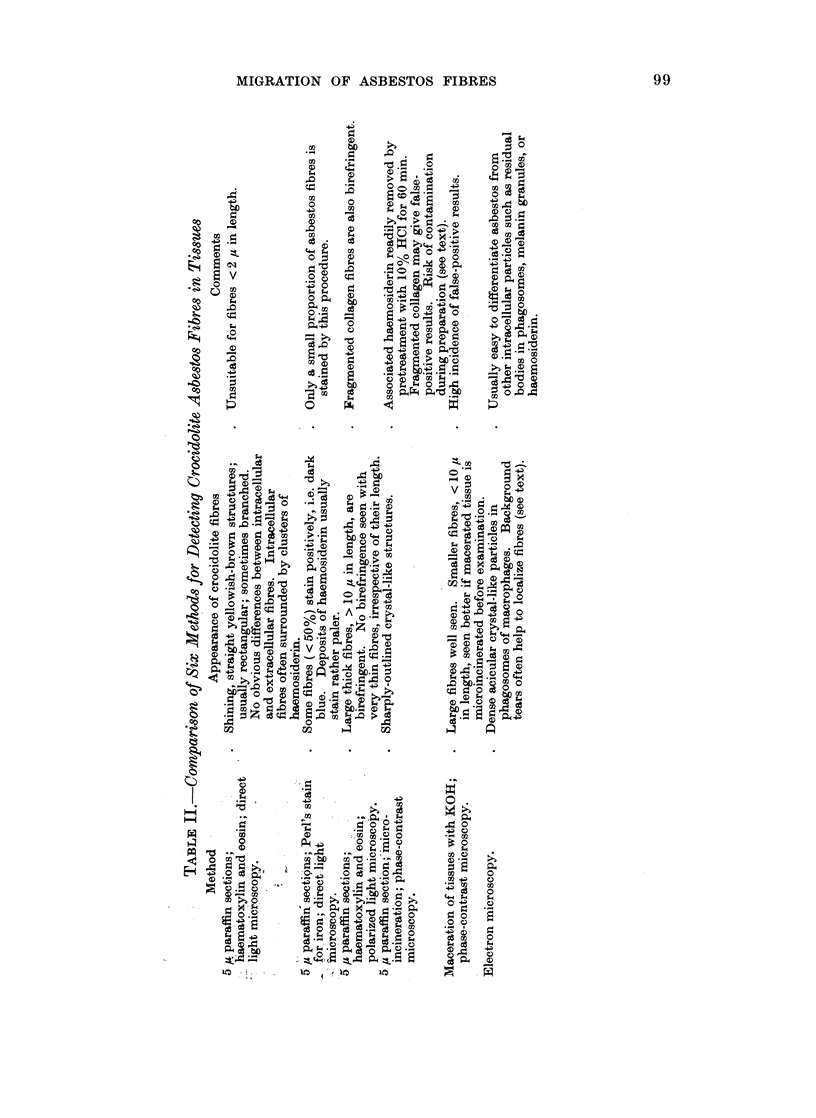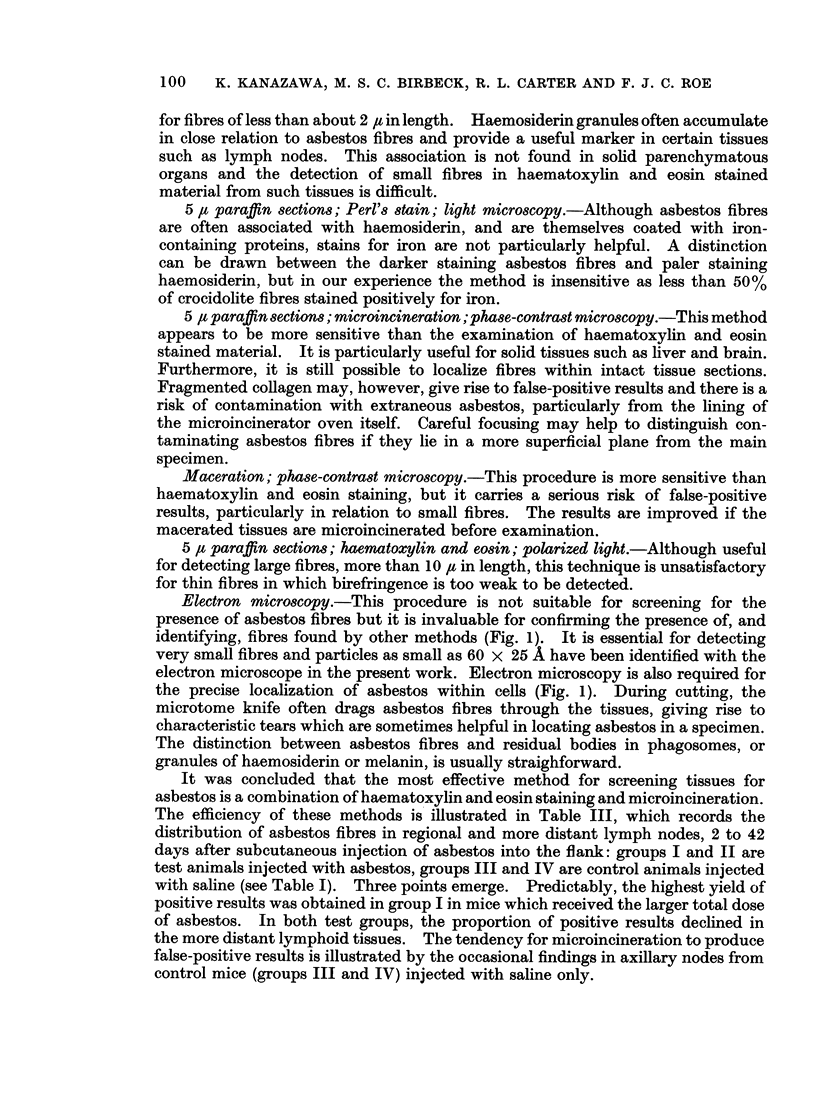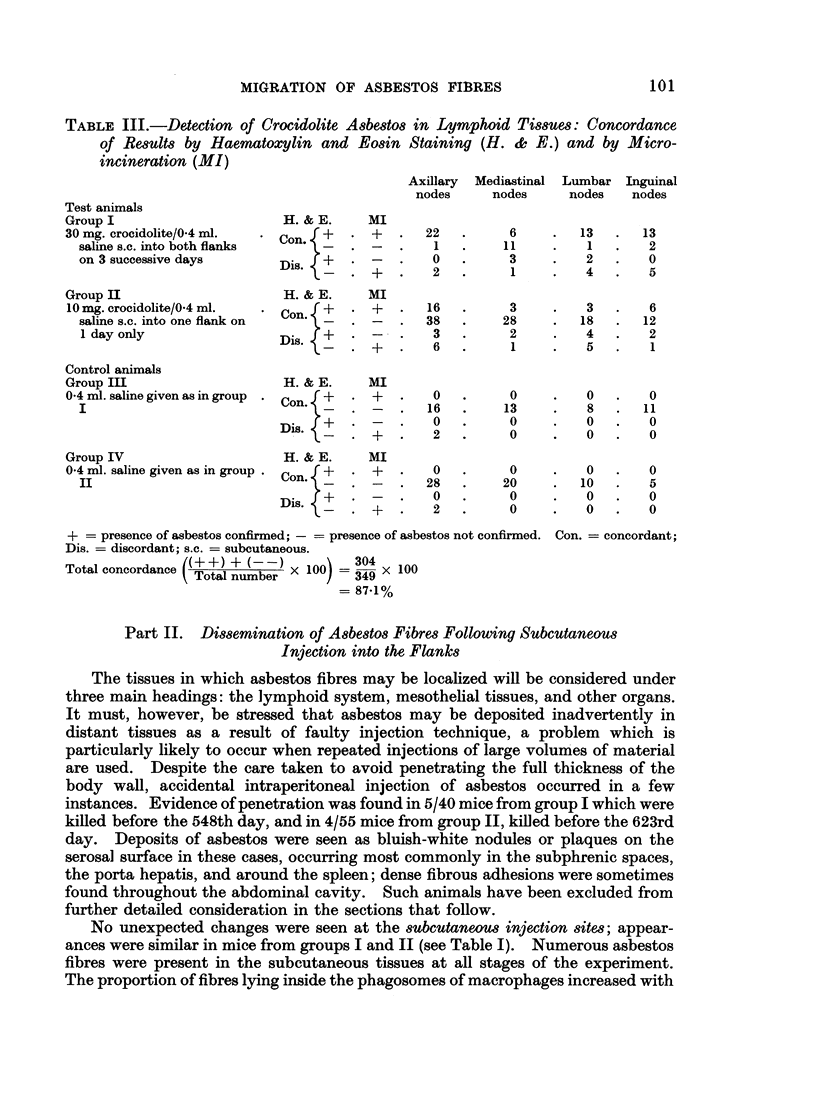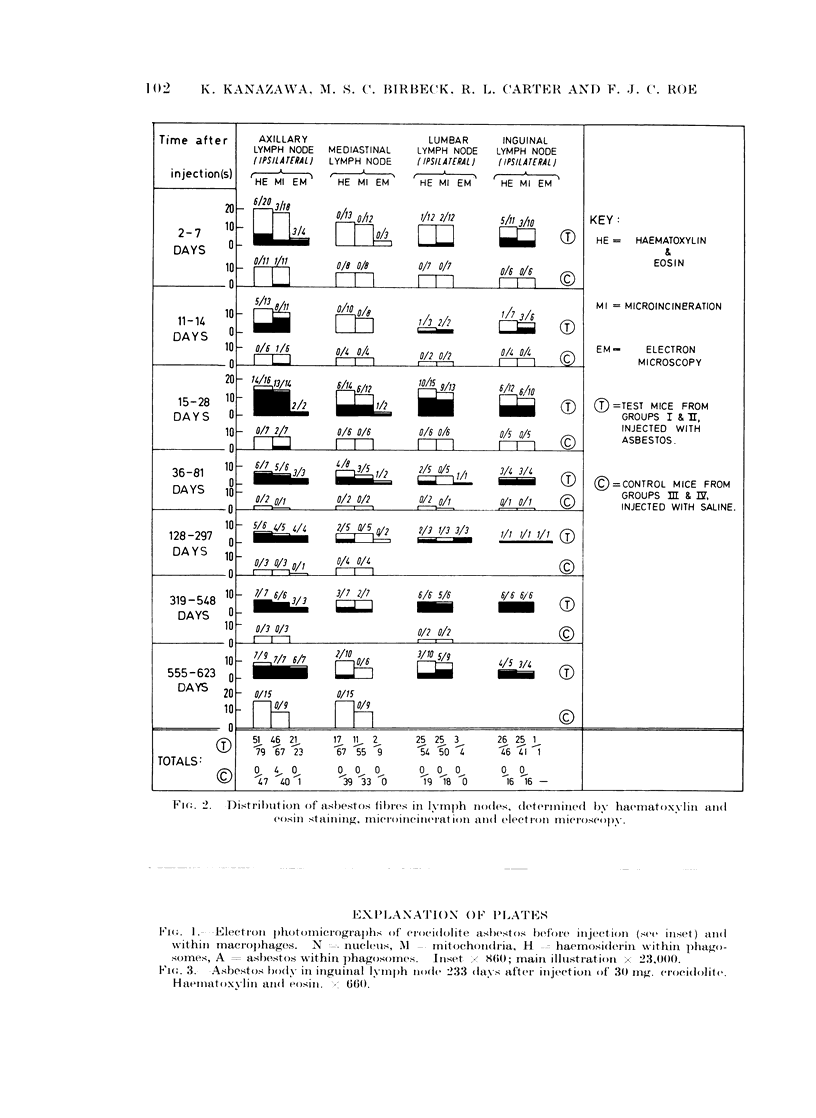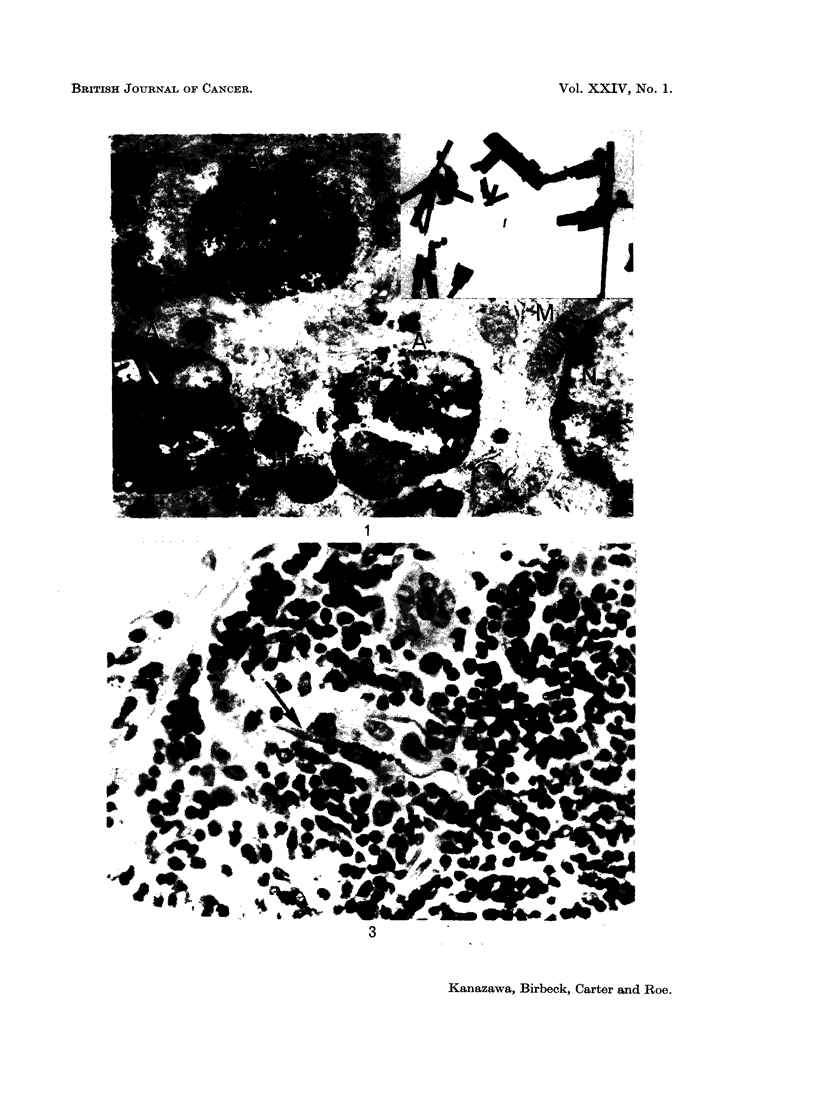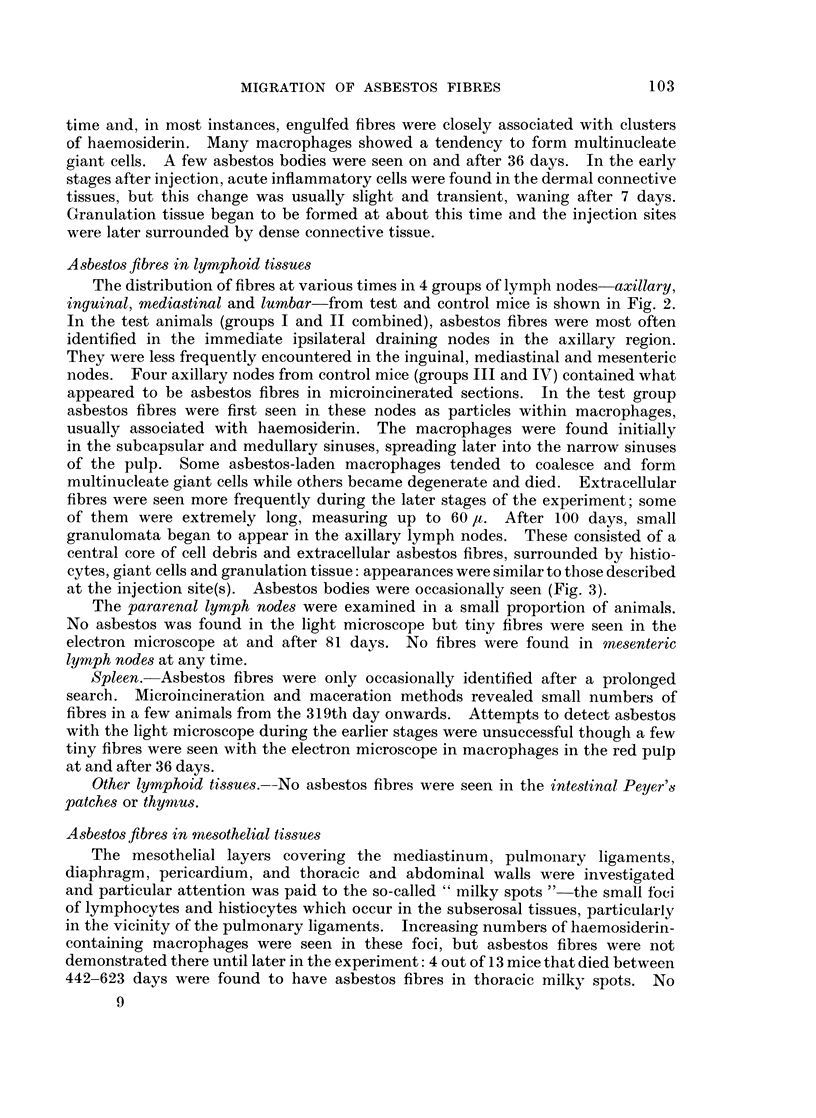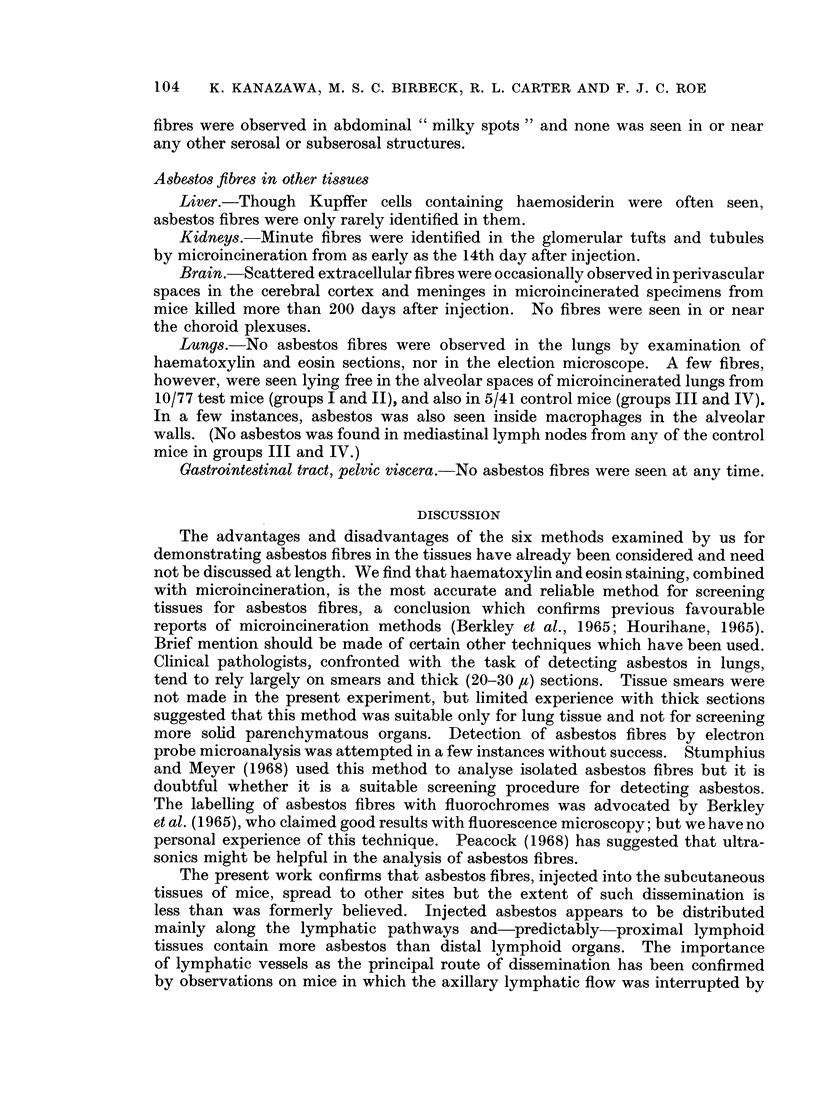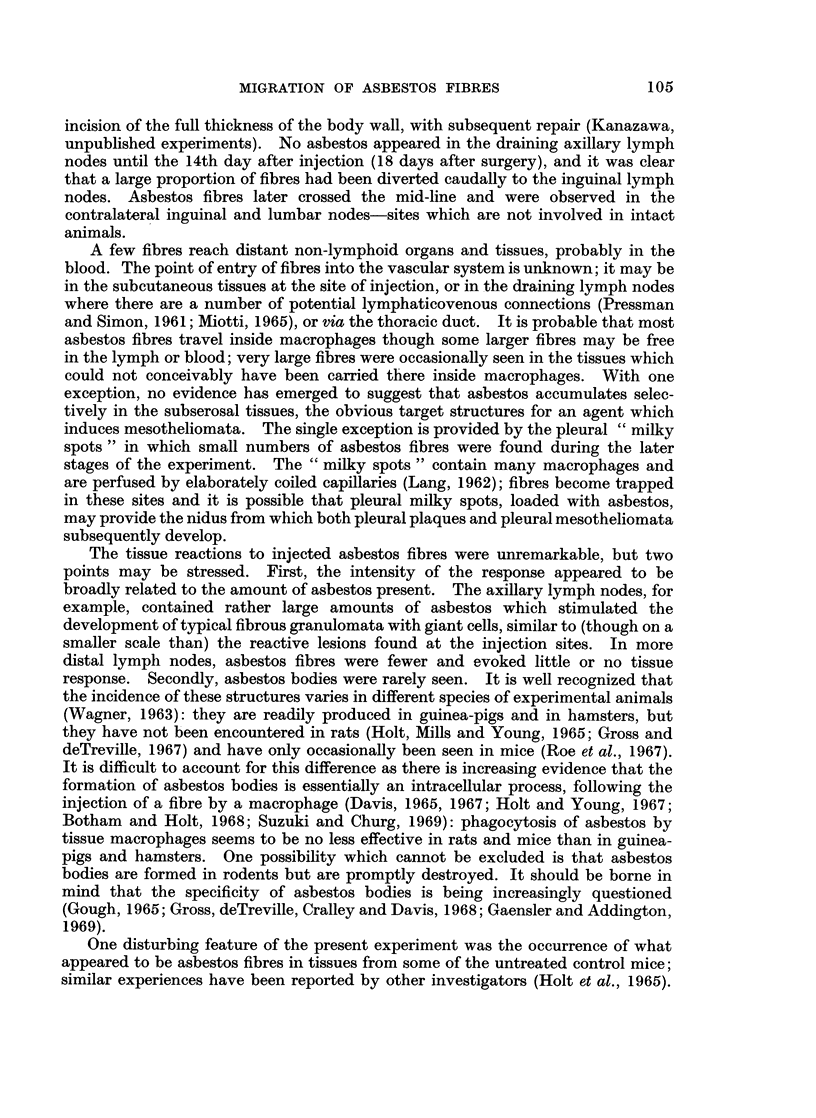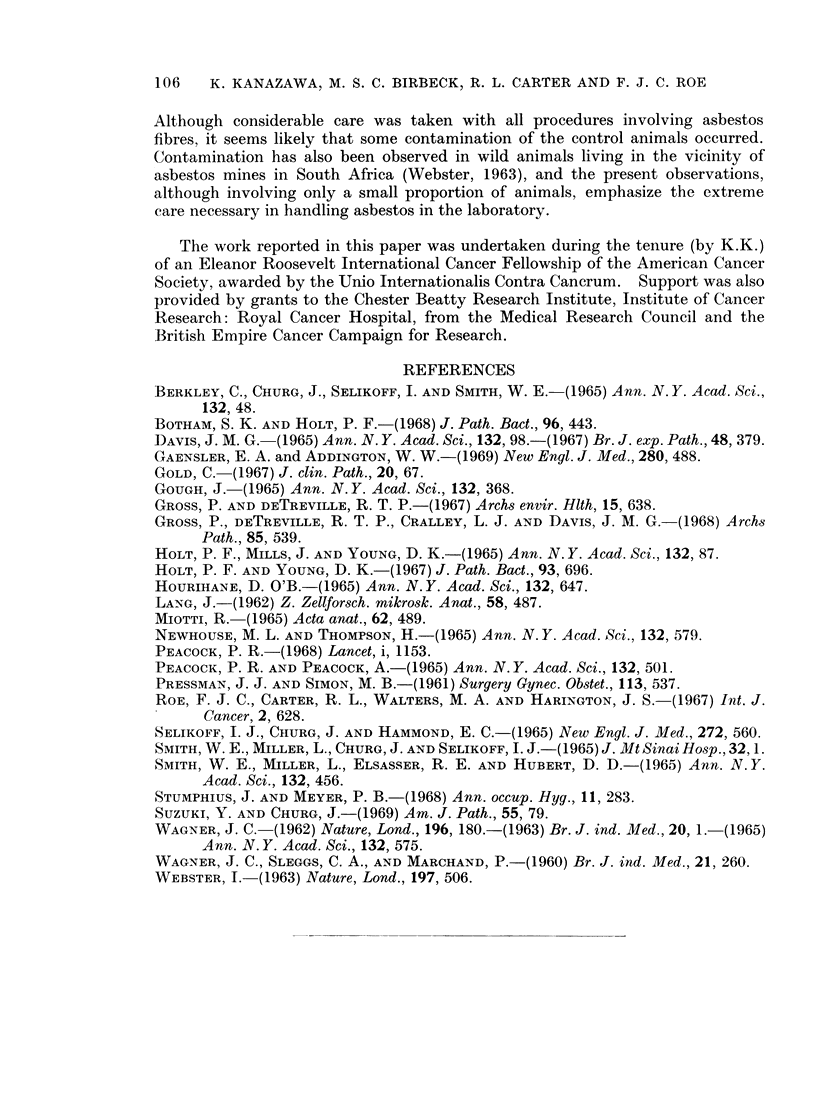# Migration of Asbestos Fibres from Subcutaneous Injection Sites in Mice

**DOI:** 10.1038/bjc.1970.13

**Published:** 1970-03

**Authors:** K. Kanazawa, M. S. C. Birbeck, R. L. Carter, F. J. C. Roe

## Abstract

**Images:**


### MIGRATION OF ASBESTOS FIBRES FROM SUBCUTANEOUS INJECTION SITES IN MICE

K. KANAZAWA, M. S. C. BIRBECK, R. L. CARTER AND F. J. C. ROE

*From the Chester Beatty Research Institute, Institute of Cancer Research: Royal Cancer Hospital, Fulham Road, London, S.W.3*

Received for publication November 11, 1969

**SUMMARY.—Crocidolite asbestos fibres, suspended in physiological saline, were injected subcutaneously into one or both flanks of 95 CBA/Lac female mice; 75 control mice received injections of saline only. Most animals were killed at chosen intervals of between 2 and 42 days after injection but some were left for longer periods of up to 623 days. At autopsy, many lymphoid and non-lymphoid structures were removed and examined for the presence of asbestos by the following techniques: haematoxylin and eosin staining followed by conventional and polarized light microscopy; Perl's stain; microincineration followed by phase-contrast microscopy; maceration with KOH followed by phase-contrast microscopy; and electron microscopy.**

**A combination of haematoxylin and eosin staining and microincineration was found to be the most convenient and reliable method for demonstrating asbestos fibres in the tissues. Electron microscopy was essential for detecting very small fibres and for locating them to specific intracellular structures.**

**The morphological findings indicate that some migration of asbestos fibres away from the initial site of injection takes place. Dissemination is usually along lymphatic pathways and fibres tend to accumulate in the lymphoid tissues, particularly in the regional (axillary) lymph nodes; smaller amounts were found in inguinal, mediastinal and lumbar nodes. The fibres were usually intracellular, lying inside the phagosomes of macrophages, but larger fibres were sometimes encountered lying free. Small numbers of fibres were seen in the spleen and also in non-lymphoid organs such as the liver, kidneys and brain —suggesting that some asbestos may enter the blood stream. There was no evidence of massive or selective spread to subserosal tissues in the thorax or abdomen, though trapping of asbestos fibres was observed in pleural " milky spots " in long-term survivors. The possible role of milky spots in the development of pleural plaques and mesotheliomata is discussed.**

THERE is increasing evidence that exposure to asbestos is associated with the development of mesotheliomata in man (Wagner, Sleggs and Marchand, 1960; Wagner, 1965; Newhouse and Thompson, 1965; Selikoff, Churg and Hammond, 1965). This association is borne out by experimental studies in animals where mesotheliomata have been induced by injecting asbestos directly into the pleural space of rats (Wagner, 1962), hamsters (Smith, Miller, Churg and Selikoff, 1965; Smith, Miller, Elsasser and Hubert, 1965) and fowls (Peacock and Peacock, 1965). Roe, Carter, Walters and Harington (1967) reported the induction of pleural and peritoneal mesotheliomata in mice after asbestos had been injected subcutaneously into the flanks—*i.e.* initially remote from the regions where mesotheliomata subsequently developed. At that time, it was suggested that asbestos fibres might spread widely from the original injection sites and find their way in a semiselective fashion to the subserosal layer of the thoracic and abdominal viscera.

An attempt has now been made to examine in more detail the dissemination of asbestos fibres after their injection into the subcutaneous tissues of mice. A prerequisite of any such study is a careful evaluation of the techniques available for detecting asbestos in the tissues, and the current results are accordingly presented in two parts—first, an appraisal of six methods for identifying asbestos fibres in the tissues, followed by an account of the anatomical distribution of these fibres after subcutaneous injection into one or both flanks of mice.

### MATERIALS AND METHODS

### *Asbestos*

A U.I.C.C. reference sample of crocidolite was supplied by Dr. J. S. Harington (South African Institute for Medical Research). The distributions of the fibre length were determined by Dr. V. Timbrell (M.R.C. Pneumoconiosis Research Unit, Llandough Hospital, Penarth, Glamorgan). Approximately 50% of the fibres were between 0·2 and 1·2 $\mu$ in length, and the remaining 50% between 1·2 and 2·0 $\mu$; less than 5% exceeded 7 $\mu$ in length. The fibres were suspended in physiological saline before injection.

### *Mice*

One hundred and seventy CBA/Lac female mice were used, aged 6–8 weeks and weighing about 25 g. They were maintained on a cubed diet (Diet No. 86, Messrs. Dixon Ltd., Ware, Herts.) and water *ad libitum*, and were kept in metal cages, 10 mice in each.

### *Conduct of experiment*

The mice were divided into 4 groups. Details of treatment are shown in Table I. Most of the animals were killed at chosen intervals between 2 and 42 days after injection. Some mice were allowed their full life span and survived for periods of up to 548 days (Group I) and 623 days (Group II).

TABLE I.—*Details of Treatment in Test and Control Groups*

| Group | No. of mice | Treatment |
|---|---|---|
| I | 40 | 30 mg. crocidolite/0·4 ml. physiological saline. Injected subcutaneously into *both* flanks on 3 successive days. |
| II | 55 | 10 mg. crocidolite/0·4 ml. physiological saline. Injected subcutaneously into R. flank only, on 1 day. |
| III | 20 | 0·4 ml. physiological saline only, given as in group I. |
| IV | 55 | 0·4 ml. physiological saline only, given as in group II. |

*Notes*

1. Mice in groups I and III were derived from a single batch allocated at random to these 2 groups. Mice in groups II and IV were derived from a separate batch, also allocated randomly between the groups.
2. A gauge No. 1 needle was used for all injections.

Animals were killed by ether vapour or by cervical dislocation. Standard necropsies were performed. Injection sites were examined for evidence of inadvertent injection of asbestos into the abdominal or thoracic cavities. Care was taken to avoid contamination of organs with asbestos during autopsy. The following tissues were removed routinely for examination: axillary, inguinal, mediastinal, lumbar and mesenteric lymph nodes; thymus, trachea and oesophagus in one block; heart and lungs in one block; liver; spleen; kidneys; ovaries; brain; gastrointestinal tract; the thoracic wall and abdominal wall, both at and away from the injection site(s).

*Methods used for detecting asbestos fibres in the tissues*

*Light microscopy.*—The tissues, fixed in Bouin's solution, were investigated as follows:

1. Paraffin sections, prepared at 5 $\mu$, were stained either with haematoxylin and eosin, or by Perl's method for iron, and examined by conventional light microscopy.
2. The haematoxylin and eosin stained sections were also examined under polarized light for the presence of birefringent particles in the tissues.
3. After dewaxing with xylol and alcohol, additional sections were incinerated at 500° C. for 1 hour in a SUNVIC microincinerator (Gallenkamp). They were then dry-mounted and examined by phase-contrast microscopy.

*Electron microscopy.*—Tissues were fixed in 3% glutaraldehyde in 1/5 M cacodylate buffer (pH 7·4) for 2 hours, postfixed with 1% osmium tetroxide in veronal acetate buffer (pH 7·4) for 30 minutes, dehydrated with methanol, treated with propylene oxide, and then embedded in araldite. Ultrathin section (600 to 900 Å) were cut on a Huxley-type ultramicrotome. Sections were stained with Karnovsky's alkaline lead staining medium and examined in a Siemens or a Philips EM 300 electron microscope. Samples of crocidolite fibres were embedded in araldite in the same way for control purposes.

*Tissue maceration.*—The residue of each specimen was minced with a fine scalpel (a new blade being used for each specimen to avoid transfer of asbestos from one tissue to the next), and then macerated by a modification of the method described by Gold (1967). About 0·5 g. of the minced tissue was mixed with 10 ml. of 40% aqueous potassium hydroxide and heated on a water bath for 1–2 hours until the tissue had been finely fragmented. The resulting suspension was then centrifuged, washed with three changes of distilled water, and a smear was prepared from the deposit. This was mounted dry for examination by phase-contrast microscopy.

### RESULTS

### Part I. *Comparison of Various Methods for the Detection of Asbestos Fibres in the Tissues*

The presence of fibres in the subcutaneous tissues at the injection-site in test mice given asbestos, and the absence of such fibres in control mice injected with saline, provided the positive and negative controls necessary for assessing the value of different methods for detecting asbestos fibres in various tissues. The main observations in relation to the six methods used are summarized in Table II. Certain general points are worth stressing.

*5 $\mu$ paraffin sections; haematoxylin and eosin; light microscopy.*—The range of asbestos fibres seen is obviously limited by their size; the method is unsuitable

TABLE II.—*Comparison of Six Methods for Detecting Crocidolite Asbestos Fibres in Tissues*

| Method | Appearance of crocidolite fibres | Comments |
|---|---|---|
| 5 μ paraffin sections; haematoxylin and eosin; direct light microscopy. | Shining, straight yellowish-brown structures; usually rectangular; sometimes branched. No obvious differences between intracellular and extracellular fibres. Intracellular fibres often surrounded by clusters of haemosiderin. | Unsuitable for fibres $< 2$ μ in length. |
| 5 μ paraffin sections; Perl's stain for iron; direct light microscopy. | Some fibres ($< 50\%$) stain positively, i.e. dark blue. Deposits of haemosiderin usually stain rather paler. | Only a small proportion of asbestos fibres is stained by this procedure. |
| 5 μ paraffin sections; haematoxylin and eosin; polarized light microscopy. | Large thick fibres, $> 10$ μ in length, are birefringent. No birefringence seen with very thin fibres, irrespective of their length. | Fragmented collagen fibres are also birefringent. |
| 5 μ paraffin section; micro-incineration; phase-contrast microscopy. | Sharply-outlined crystal-like structures. | Associated haemosiderin readily removed by pretreatment with 10% HCl for 60 min. Fragmented collagen may give false-positive results. Risk of contamination during preparation (see text). |
| Maceration of tissues with KOH; phase-contrast microscopy. | Large fibres well seen. Smaller fibres, $< 10$ μ in length, seen better if macerated tissue is microincinerated before examination. | High incidence of false-positive results. |
| Electron microscopy. | Dense acicular crystal-like particles in phagosomes of macrophages. Background tears often help to localize fibres (see text). | Usually easy to differentiate asbestos from other intracellular particles such as residual bodies in phagosomes, melanin granules, or haemosiderin. |

for fibres of less than about 2 $\mu$ in length. Haemosiderin granules often accumulate in close relation to asbestos fibres and provide a useful marker in certain tissues such as lymph nodes. This association is not found in solid parenchymatous organs and the detection of small fibres in haematoxylin and eosin stained material from such tissues is difficult.

5 $\mu$ *paraffin sections; Perl's stain; light microscopy.*—Although asbestos fibres are often associated with haemosiderin, and are themselves coated with iron-containing proteins, stains for iron are not particularly helpful. A distinction can be drawn between the darker staining asbestos fibres and paler staining haemosiderin, but in our experience the method is insensitive as less than 50% of crocidolite fibres stained positively for iron.

5 $\mu$ *paraffin sections; microincineration; phase-contrast microscopy.*—This method appears to be more sensitive than the examination of haematoxylin and eosin stained material. It is particularly useful for solid tissues such as liver and brain. Furthermore, it is still possible to localize fibres within intact tissue sections. Fragmented collagen may, however, give rise to false-positive results and there is a risk of contamination with extraneous asbestos, particularly from the lining of the microincinerator oven itself. Careful focusing may help to distinguish contaminating asbestos fibres if they lie in a more superficial plane from the main specimen.

*Maceration; phase-contrast microscopy.*—This procedure is more sensitive than haematoxylin and eosin staining, but it carries a serious risk of false-positive results, particularly in relation to small fibres. The results are improved if the macerated tissues are microincinerated before examination.

5 $\mu$ *paraffin sections; haematoxylin and eosin; polarized light.*—Although useful for detecting large fibres, more than 10 $\mu$ in length, this technique is unsatisfactory for thin fibres in which birefringence is too weak to be detected.

*Electron microscopy.*—This procedure is not suitable for screening for the presence of asbestos fibres but it is invaluable for confirming the presence of, and identifying, fibres found by other methods (Fig. 1). It is essential for detecting very small fibres and particles as small as 60 × 25 Å have been identified with the electron microscope in the present work. Electron microscopy is also required for the precise localization of asbestos within cells (Fig. 1). During cutting, the microtome knife often drags asbestos fibres through the tissues, giving rise to characteristic tears which are sometimes helpful in locating asbestos in a specimen. The distinction between asbestos fibres and residual bodies in phagosomes, or granules of haemosiderin or melanin, is usually straighforward.

It was concluded that the most effective method for screening tissues for asbestos is a combination of haematoxylin and eosin staining and microincineration. The efficiency of these methods is illustrated in Table III, which records the distribution of asbestos fibres in regional and more distant lymph nodes, 2 to 42 days after subcutaneous injection of asbestos into the flank: groups I and II are test animals injected with asbestos, groups III and IV are control animals injected with saline (see Table I). Three points emerge. Predictably, the highest yield of positive results was obtained in group I in mice which received the larger total dose of asbestos. In both test groups, the proportion of positive results declined in the more distant lymphoid tissues. The tendency for microincineration to produce false-positive results is illustrated by the occasional findings in axillary nodes from control mice (groups III and IV) injected with saline only.

TABLE III.—*Detection of Crocidolite Asbestos in Lymphoid Tissues: Concordance of Results by Haematoxylin and Eosin Staining (H. & E.) and by Micro-incineration (MI)*

| | H. & E. | MI | Axillary nodes | Mediastinal nodes | Lumbar nodes | Inguinal nodes |
|---|---|---|---|---|---|---|
| Test animals | | | | | | |
| Group I | | | | | | |
| 30 mg. crocidolite/0·4 ml. saline s.c. into both flanks on 3 successive days | Con. + | + | 22 | 6 | 13 | 13 |
| | Con. − | − | 1 | 11 | 1 | 2 |
| | Dis. + | − | 0 | 3 | 2 | 0 |
| | Dis. − | + | 2 | 1 | 4 | 5 |
| Group II | | | | | | |
| 10 mg. crocidolite/0·4 ml. saline s.c. into one flank on 1 day only | Con. + | + | 16 | 3 | 3 | 6 |
| | Con. − | − | 38 | 28 | 18 | 12 |
| | Dis. + | − | 3 | 2 | 4 | 2 |
| | Dis. − | + | 6 | 1 | 5 | 1 |
| Control animals | | | | | | |
| Group III | | | | | | |
| 0·4 ml. saline given as in group I | Con. + | + | 0 | 0 | 0 | 0 |
| | Con. − | − | 16 | 13 | 8 | 11 |
| | Dis. + | − | 0 | 0 | 0 | 0 |
| | Dis. − | + | 2 | 0 | 0 | 0 |
| Group IV | | | | | | |
| 0·4 ml. saline given as in group II | Con. + | + | 0 | 0 | 0 | 0 |
| | Con. − | − | 28 | 20 | 10 | 5 |
| | Dis. + | − | 0 | 0 | 0 | 0 |
| | Dis. − | + | 2 | 0 | 0 | 0 |

\+ = presence of asbestos confirmed; − = presence of asbestos not confirmed. Con. = concordant; Dis. = discordant; s.c. = subcutaneous.

$$\text{Total concordance}\left(\frac{(++)+(--)}{\text{Total number}} \times 100\right) = \frac{304}{349} \times 100 = 87{\cdot}1\%$$

### Part II. *Dissemination of Asbestos Fibres Following Subcutaneous Injection into the Flanks*

The tissues in which asbestos fibres may be localized will be considered under three main headings: the lymphoid system, mesothelial tissues, and other organs. It must, however, be stressed that asbestos may be deposited inadvertently in distant tissues as a result of faulty injection technique, a problem which is particularly likely to occur when repeated injections of large volumes of material are used. Despite the care taken to avoid penetrating the full thickness of the body wall, accidental intraperitoneal injection of asbestos occurred in a few instances. Evidence of penetration was found in 5/40 mice from group I which were killed before the 548th day, and in 4/55 mice from group II, killed before the 623rd day. Deposits of asbestos were seen as bluish-white nodules or plaques on the serosal surface in these cases, occurring most commonly in the subphrenic spaces, the porta hepatis, and around the spleen; dense fibrous adhesions were sometimes found throughout the abdominal cavity. Such animals have been excluded from further detailed consideration in the sections that follow.

No unexpected changes were seen at the *subcutaneous injection sites*; appearances were similar in mice from groups I and II (see Table I). Numerous asbestos fibres were present in the subcutaneous tissues at all stages of the experiment. The proportion of fibres lying inside the phagosomes of macrophages increased with

FIG. 2.—Distribution of asbestos fibres in lymph nodes, determined by haematoxylin and eosin staining, microincineration and electron microscopy.

### EXPLANATION OF PLATES

FIG. 1.—Electron photomicrographs of crocidolite asbestos before injection (see inset) and within macrophages. N = nucleus, M = mitochondria, H = haemosiderin within phagosomes, A = asbestos within phagosomes. Inset × 860; main illustration × 23,000.

FIG. 3.—Asbestos body in inguinal lymph node 233 days after injection of 30 mg. crocidolite. Haematoxylin and eosin. × 660.

1

3

time and, in most instances, engulfed fibres were closely associated with clusters of haemosiderin. Many macrophages showed a tendency to form multinucleate giant cells. A few asbestos bodies were seen on and after 36 days. In the early stages after injection, acute inflammatory cells were found in the dermal connective tissues, but this change was usually slight and transient, waning after 7 days. Granulation tissue began to be formed at about this time and the injection sites were later surrounded by dense connective tissue.

*Asbestos fibres in lymphoid tissues*

The distribution of fibres at various times in 4 groups of lymph nodes—*axillary, inguinal, mediastinal* and *lumbar*—from test and control mice is shown in Fig. 2. In the test animals (groups I and II combined), asbestos fibres were most often identified in the immediate ipsilateral draining nodes in the axillary region. They were less frequently encountered in the inguinal, mediastinal and mesenteric nodes. Four axillary nodes from control mice (groups III and IV) contained what appeared to be asbestos fibres in microincinerated sections. In the test group asbestos fibres were first seen in these nodes as particles within macrophages, usually associated with haemosiderin. The macrophages were found initially in the subcapsular and medullary sinuses, spreading later into the narrow sinuses of the pulp. Some asbestos-laden macrophages tended to coalesce and form multinucleate giant cells while others became degenerate and died. Extracellular fibres were seen more frequently during the later stages of the experiment; some of them were extremely long, measuring up to 60 $\mu$. After 100 days, small granulomata began to appear in the axillary lymph nodes. These consisted of a central core of cell debris and extracellular asbestos fibres, surrounded by histiocytes, giant cells and granulation tissue: appearances were similar to those described at the injection site(s). Asbestos bodies were occasionally seen (Fig. 3).

The *pararenal lymph nodes* were examined in a small proportion of animals. No asbestos was found in the light microscope but tiny fibres were seen in the electron microscope at and after 81 days. No fibres were found in *mesenteric lymph nodes* at any time.

*Spleen.*—Asbestos fibres were only occasionally identified after a prolonged search. Microincineration and maceration methods revealed small numbers of fibres in a few animals from the 319th day onwards. Attempts to detect asbestos with the light microscope during the earlier stages were unsuccessful though a few tiny fibres were seen with the electron microscope in macrophages in the red pulp at and after 36 days.

*Other lymphoid tissues.*—No asbestos fibres were seen in the *intestinal Peyer's patches* or *thymus.*

*Asbestos fibres in mesothelial tissues*

The mesothelial layers covering the mediastinum, pulmonary ligaments, diaphragm, pericardium, and thoracic and abdominal walls were investigated and particular attention was paid to the so-called " milky spots "—the small foci of lymphocytes and histiocytes which occur in the subserosal tissues, particularly in the vicinity of the pulmonary ligaments. Increasing numbers of haemosiderin-containing macrophages were seen in these foci, but asbestos fibres were not demonstrated there until later in the experiment: 4 out of 13 mice that died between 442–623 days were found to have asbestos fibres in thoracic milky spots. No fibres were observed in abdominal " milky spots " and none was seen in or near any other serosal or subserosal structures.

*Asbestos fibres in other tissues*

*Liver.*—Though Kupffer cells containing haemosiderin were often seen, asbestos fibres were only rarely identified in them.

*Kidneys.*—Minute fibres were identified in the glomerular tufts and tubules by microincineration from as early as the 14th day after injection.

*Brain.*—Scattered extracellular fibres were occasionally observed in perivascular spaces in the cerebral cortex and meninges in microincinerated specimens from mice killed more than 200 days after injection. No fibres were seen in or near the choroid plexuses.

*Lungs.*—No asbestos fibres were observed in the lungs by examination of haematoxylin and eosin sections, nor in the election microscope. A few fibres, however, were seen lying free in the alveolar spaces of microincinerated lungs from 10/77 test mice (groups I and II), and also in 5/41 control mice (groups III and IV). In a few instances, asbestos was also seen inside macrophages in the alveolar walls. (No asbestos was found in mediastinal lymph nodes from any of the control mice in groups III and IV.)

*Gastrointestinal tract, pelvic viscera.*—No asbestos fibres were seen at any time.

### DISCUSSION

The advantages and disadvantages of the six methods examined by us for demonstrating asbestos fibres in the tissues have already been considered and need not be discussed at length. We find that haematoxylin and eosin staining, combined with microincineration, is the most accurate and reliable method for screening tissues for asbestos fibres, a conclusion which confirms previous favourable reports of microincineration methods (Berkley *et al.*, 1965; Hourihane, 1965). Brief mention should be made of certain other techniques which have been used. Clinical pathologists, confronted with the task of detecting asbestos in lungs, tend to rely largely on smears and thick (20–30 $\mu$) sections. Tissue smears were not made in the present experiment, but limited experience with thick sections suggested that this method was suitable only for lung tissue and not for screening more solid parenchymatous organs. Detection of asbestos fibres by electron probe microanalysis was attempted in a few instances without success. Stumphius and Meyer (1968) used this method to analyse isolated asbestos fibres but it is doubtful whether it is a suitable screening procedure for detecting asbestos. The labelling of asbestos fibres with fluorochromes was advocated by Berkley *et al.* (1965), who claimed good results with fluorescence microscopy; but we have no personal experience of this technique. Peacock (1968) has suggested that ultrasonics might be helpful in the analysis of asbestos fibres.

The present work confirms that asbestos fibres, injected into the subcutaneous tissues of mice, spread to other sites but the extent of such dissemination is less than was formerly believed. Injected asbestos appears to be distributed mainly along the lymphatic pathways and—predictably—proximal lymphoid tissues contain more asbestos than distal lymphoid organs. The importance of lymphatic vessels as the principal route of dissemination has been confirmed by observations on mice in which the axillary lymphatic flow was interrupted by incision of the full thickness of the body wall, with subsequent repair (Kanazawa, unpublished experiments). No asbestos appeared in the draining axillary lymph nodes until the 14th day after injection (18 days after surgery), and it was clear that a large proportion of fibres had been diverted caudally to the inguinal lymph nodes. Asbestos fibres later crossed the mid-line and were observed in the contralateral inguinal and lumbar nodes—sites which are not involved in intact animals.

A few fibres reach distant non-lymphoid organs and tissues, probably in the blood. The point of entry of fibres into the vascular system is unknown; it may be in the subcutaneous tissues at the site of injection, or in the draining lymph nodes where there are a number of potential lymphaticovenous connections (Pressman and Simon, 1961; Miotti, 1965), or *via* the thoracic duct. It is probable that most asbestos fibres travel inside macrophages though some larger fibres may be free in the lymph or blood; very large fibres were occasionally seen in the tissues which could not conceivably have been carried there inside macrophages. With one exception, no evidence has emerged to suggest that asbestos accumulates selectively in the subserosal tissues, the obvious target structures for an agent which induces mesotheliomata. The single exception is provided by the pleural " milky spots " in which small numbers of asbestos fibres were found during the later stages of the experiment. The " milky spots " contain many macrophages and are perfused by elaborately coiled capillaries (Lang, 1962); fibres become trapped in these sites and it is possible that pleural milky spots, loaded with asbestos, may provide the nidus from which both pleural plaques and pleural mesotheliomata subsequently develop.

The tissue reactions to injected asbestos fibres were unremarkable, but two points may be stressed. First, the intensity of the response appeared to be broadly related to the amount of asbestos present. The axillary lymph nodes, for example, contained rather large amounts of asbestos which stimulated the development of typical fibrous granulomata with giant cells, similar to (though on a smaller scale than) the reactive lesions found at the injection sites. In more distal lymph nodes, asbestos fibres were fewer and evoked little or no tissue response. Secondly, asbestos bodies were rarely seen. It is well recognized that the incidence of these structures varies in different species of experimental animals (Wagner, 1963): they are readily produced in guinea-pigs and in hamsters, but they have not been encountered in rats (Holt, Mills and Young, 1965; Gross and deTreville, 1967) and have only occasionally been seen in mice (Roe *et al.*, 1967). It is difficult to account for this difference as there is increasing evidence that the formation of asbestos bodies is essentially an intracellular process, following the injection of a fibre by a macrophage (Davis, 1965, 1967; Holt and Young, 1967; Botham and Holt, 1968; Suzuki and Churg, 1969): phagocytosis of asbestos by tissue macrophages seems to be no less effective in rats and mice than in guinea-pigs and hamsters. One possibility which cannot be excluded is that asbestos bodies are formed in rodents but are promptly destroyed. It should be borne in mind that the specificity of asbestos bodies is being increasingly questioned (Gough, 1965; Gross, deTreville, Cralley and Davis, 1968; Gaensler and Addington, 1969).

One disturbing feature of the present experiment was the occurrence of what appeared to be asbestos fibres in tissues from some of the untreated control mice; similar experiences have been reported by other investigators (Holt *et al.*, 1965).

Although considerable care was taken with all procedures involving asbestos fibres, it seems likely that some contamination of the control animals occurred. Contamination has also been observed in wild animals living in the vicinity of asbestos mines in South Africa (Webster, 1963), and the present observations, although involving only a small proportion of animals, emphasize the extreme care necessary in handling asbestos in the laboratory.

The work reported in this paper was undertaken during the tenure (by K.K.) of an Eleanor Roosevelt International Cancer Fellowship of the American Cancer Society, awarded by the Unio Internationalis Contra Cancrum. Support was also provided by grants to the Chester Beatty Research Institute, Institute of Cancer Research: Royal Cancer Hospital, from the Medical Research Council and the British Empire Cancer Campaign for Research.

### REFERENCES

Berkley, C., Churg, J., Selikoff, I. and Smith, W. E.—(1965) *Ann. N.Y. Acad. Sci.*, **132**, 48.

Botham, S. K. and Holt, P. F.—(1968) *J. Path. Bact.*, **96**, 443.

Davis, J. M. G.—(1965) *Ann. N.Y. Acad. Sci.*, **132**, 98.—(1967) *Br. J. exp. Path.*, **48**, 379.

Gaensler, E. A. and Addington, W. W.—(1969) *New Engl. J. Med.*, **280**, 488.

Gold, C.—(1967) *J. clin. Path.*, **20**, 67.

Gough, J.—(1965) *Ann. N.Y. Acad. Sci.*, **132**, 368.

Gross, P. and deTreville, R. T. P.—(1967) *Archs envir. Hlth*, **15**, 638.

Gross, P., deTreville, R. T. P., Cralley, L. J. and Davis, J. M. G.—(1968) *Archs Path.*, **85**, 539.

Holt, P. F., Mills, J. and Young, D. K.—(1965) *Ann. N.Y. Acad. Sci.*, **132**, 87.

Holt, P. F. and Young, D. K.—(1967) *J. Path. Bact.*, **93**, 696.

Hourihane, D. O'B.—(1965) *Ann. N.Y. Acad. Sci.*, **132**, 647.

Lang, J.—(1962) *Z. Zellforsch. mikrosk. Anat.*, **58**, 487.

Miotti, R.—(1965) *Acta anat.*, **62**, 489.

Newhouse, M. L. and Thompson, H.—(1965) *Ann. N.Y. Acad. Sci.*, **132**, 579.

Peacock, P. R.—(1968) *Lancet*, i, 1153.

Peacock, P. R. and Peacock, A.—(1965) *Ann. N.Y. Acad. Sci.*, **132**, 501.

Pressman, J. J. and Simon, M. B.—(1961) *Surgery Gynec. Obstet.*, **113**, 537.

Roe, F. J. C., Carter, R. L., Walters, M. A. and Harington, J. S.—(1967) *Int. J. Cancer*, **2**, 628.

Selikoff, I. J., Churg, J. and Hammond, E. C.—(1965) *New Engl. J. Med.*, **272**, 560.

Smith, W. E., Miller, L., Churg, J. and Selikoff, I. J.—(1965) *J. Mt Sinai Hosp.*, **32**, 1.

Smith, W. E., Miller, L., Elsasser, R. E. and Hubert, D. D.—(1965) *Ann. N.Y. Acad. Sci.*, **132**, 456.

Stumphius, J. and Meyer, P. B.—(1968) *Ann. occup. Hyg.*, **11**, 283.

Suzuki, Y. and Churg, J.—(1969) *Am. J. Path.*, **55**, 79.

Wagner, J. C.—(1962) *Nature, Lond.*, **196**, 180.—(1963) *Br. J. ind. Med.*, **20**, 1.—(1965) *Ann. N.Y. Acad. Sci.*, **132**, 575.

Wagner, J. C., Sleggs, C. A., and Marchand, P.—(1960) *Br. J. ind. Med.*, **21**, 260.

Webster, I.—(1963) *Nature, Lond.*, **197**, 506.